# A Novel circRNA–miRNA–mRNA Hub Regulatory Network in Lung Adenocarcinoma

**DOI:** 10.3389/fgene.2021.673501

**Published:** 2021-07-07

**Authors:** Haiwei Zuo, Xia Li, Xixi Zheng, Qiuwen Sun, Qianqian Yang, Yong Xin

**Affiliations:** ^1^School of Medical Information and Engineering, Xuzhou Medical University, Xuzhou, China; ^2^Department of Radiation Oncology, Affiliated Hospital of Xuzhou Medical University, Xuzhou, China; ^3^Cancer Institute of Xuzhou Medical University, Xuzhou, China; ^4^School of Medical Imaging, Xuzhou Medical University, Xuzhou, China; ^5^Neonatal Intensive Care Unit, Affiliated Hospital of Xuzhou Medical University, Xuzhou, China

**Keywords:** lung adenocarcinoma, circRNA, ceRNA, GEO, TCGA, qRT-PCR

## Abstract

The growing evidence suggests that circular RNAs (circRNAs) have significant associations with tumor occurrence and progression, yet the regulatory mechanism of circRNAs in lung adenocarcinoma (LUAD) remains unclear. This study clarified the potentially regulatory network and functional mechanism of circRNAs in LUAD. The expression data of circRNAs, microRNAs (miRNAs), and messenger RNAs (mRNAs) were obtained from the Gene Expression Omnibus (GEO) database. Relying on GSE101586, GSE101684, and GSE112214, we identified differentially expressed circRNAs (DEcircRNAs). Depending on GSE135918 and GSE32863, we screened out differentially expressed miRNAs (DEmiRNAs) and mRNAs (DEmRNAs), respectively. Then, a novel competing endogenous RNA (ceRNA) regulatory network related to LUAD was constructed. We also revealed biological processes and signal pathways regulated by these DEcircRNAs. Based on gene expression data and survival information of LUAD patients in The Cancer Genome Atlas (TCGA) and GEO, we implemented survival analysis to select DEmRNAs related to prognosis and build a novel circRNA–miRNA–mRNA hub regulatory network. Meanwhile, quantitative real-time PCR (qRT-PCR) was utilized to validate DEcircRNAs in the ceRNA hub regulatory network. As a result, a total of 8 DEcircRNAs, 19 DEmiRNAs, and 85 DEmRNAs were identified. The novel ceRNA regulatory network included 5 circRNAs, 8 miRNAs, and 22 mRNAs. The final ceRNA hub regulatory network contained two circRNAs, two miRNAs, and two mRNAs. Gene Ontology (GO) and Kyoto Encyclopedia of Genes and Genomes (KEGG) analyses indicated that the five DEcircRNAs may affect LUAD onset and progression through Wnt signaling pathway and Hippo signaling pathway. All in all, this study revealed the regulatory network and functional mechanism of circRNA-related ceRNAs in LUAD.

## Introduction

Among all cancers, lung cancer has become the most commonly diagnosed cancer (11.6% of the total cases) and a major cause of cancer deaths (18.4% of the total cancer deaths) (Bray et al., 2018). Besides, most lung cancer patients suffered from lung adenocarcinoma (LUAD). The American Cancer Society (ACS) estimates that, in 2021, there will be 1,898,160 new cancer cases and 608,570 new cancer deaths in the United States (Siegel et al., 2021). A large quantity of evidence show that cancer has become one of the thorny public health problems across the world. In recent years, in spite of the great advances in surgical techniques, chemotherapy, radiotherapy, and immunotherapy, the overall survival (OS) and prognosis of LUAD patients remain poor (Lou et al., 2021). Therefore, it is essential for the development of diagnostic and prognostic biomarkers and effective therapeutic targets to elucidate the molecular network and regulation mechanism of LUAD.

Circular RNAs (circRNAs), a kind of RNA with a more stable structure, have been reported to act as a sponge for microRNAs (miRNAs) and interact with miRNAs, reducing the ability of miRNAs to target genes, thereby regulating gene expression (Li et al., 2015). In view of this, the role and regulatory mechanism of circRNAs in tumorigenesis and tumor progression have arose wide attention among related researchers. Recent studies have revealed the function and mechanism of some circRNAs in regulating lung cancer. First, Li et al. (2020) found that hsa_circ_0030998 could affect the function of miR-558, thereby inhibiting the proliferation, migration, invasion, and taxol resistance of lung tumor cells. In non-small cell lung cancer (NSCLC), Lu M. et al. (2020) presented that circRACGAP1 could affect the tumorigenesis and tumor progression of NSCLC by means of the circRACGAP1/miR-144-5p/CDKL1 axis. Similarly, Zhang Y. et al. (2020) illustrated that circDENND2A promoted NSCLC progression through miR-34a/CCNE1 regulatory axis. Later, He et al. (2020) declared that the expression level of plasma exo-hsa_circRNA_0056616 was higher in LUAD patients with lymph node metastasis than that in LUAD patients without lymph node metastasis. This suggested that plasma exo-hsa_circRNA_0056616 might be a biomarker to predict lymph node metastasis in LUAD patients. Although some progress has been made in the research for circRNAs, studies on circRNAs in regulating LUAD occurrence and progression remain few, and there is a lot of room for improvement.

**FIGURE 1:**
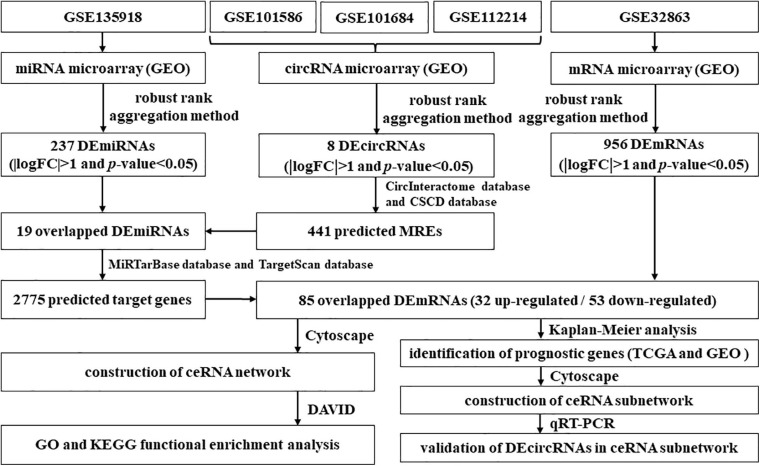
Flowchart of the comprehensive analysis process and all methods utilized in present study.

In this study, we first collected the expression profiles of circRNAs, miRNAs, and messenger RNAs (mRNAs) in LUAD from the TCGA and GEO databases, respectively. Following, based on the expression profile data, DEcircRNAs, DEmiRNAs, and DEmRNAs were identified. At the same time, we obtained circRNA–miRNA pairs and miRNA–mRNA pairs. Then, we constructed a novel circRNA–miRNA–mRNA regulatory network related to LUAD through visualizing circRNA–miRNA pairs and miRNA–mRNA pairs. In addition, we explored the biological processes and signal pathways involved in these mRNAs in the novel regulatory network via utilizing GO and KEGG enrichment analysis. For the sake of further clarifying the regulatory mechanism of circRNAs on LUAD, we downloaded comprehensive data of LUAD patients from the TCGA and GEO databases. On basis of the comprehensive data, we screened mRNAs related to the prognosis of LUAD from the mRNAs in the novel regulatory network and constructed a novel circRNA–miRNA–mRNA hub regulatory network. Finally, based on fresh frozen tissue specimen cohort, we performed quantitative real-time PCR (qRT-PCR) to validate the DEcircRNAs in the novel competing endogenous RNA (ceRNA) hub regulatory network. On the one hand, our study is beneficial to exploring diagnostic and prognostic biomarkers of LUAD. On the other hand, this study puts forward novel insights on the occurrence and progression mechanism of LUAD. The flowchart of this study is shown in Figure 1.

## Materials and Methods

### Data Acquisition From Publicly Available Databases

Six microarray datasets (GSE101586, GSE101684, GSE112214, GSE135918, GSE32863, and GSE26939) were achieved from the GEO database^1^. Specifically, GSE101586, GSE101684, and GSE112214 contain expression data of circRNAs between 12 LUAD samples and 12 normal samples. GSE135918 contains expression data of miRNAs between five LUAD samples and five normal samples. Expression data of mRNAs are included in GSE32863 between 58 LUAD samples and 58 normal samples. GSE26939 contains expression data of mRNAs and clinical information associated with 116 LUAD patients. Besides, we also acquired a total of 1,038 LUAD samples with expression profiles and complete clinical information from the TCGA database^2^. Because all data were downloaded from publicly available databases (GEO and TCGA), the approval by the Ethics Committee was not necessary.

### Fresh Frozen Tissue Specimen Cohort

The 39 pairs of fresh frozen LUAD tissues and adjacent non-tumor tissues were collected at the Affiliated Hospital of Xuzhou Medical University. These samples were preserved at −80°C for qRT-PCR. The project was granted approval by the Ethics Committee of the Affiliated Hospital of Xuzhou Medical University.

### Identification of Differentially Expressed circRNAs, miRNAs, and mRNAs

First, we adopted the normalization and logarithmic method to preprocess the raw microarray data (GSE101586, GSE101684, GSE112214, GSE135918, and GSE32863). Then, based on the Bioconductor Limma package, we integrated preprocessed microarray data (GSE101586, GSE101684, and GSE112214) to identify DEcircRNAs with thresholds of | log2(fold change)| >1 and *p* < 0.05. Simultaneously, we utilized robust rank aggregation method to rank all of the DEcircRNAs. On the basis of preprocessed microarray data (GSE135918 and GSE32863), we also obtained DEmiRNAs and DEmRNAs, respectively. In addition, the ggplot2 package was applied to visualize DEcircRNAs, DEmiRNAs, and DEmRNAs.

### Prediction of miRNA Binding Sites and miRNA Target Genes

In this study, we predicted miRNA binding sites (MREs) through integrating the Circular RNA Interactome (CircInteractome) database^3^ (Lai et al., 2020) and the Cancer-Specific CircRNA (CSCD) database^4^ (Abramowicz and Story, 2020). In detail, we treated the overlapped miRNAs between the CircInteractome database and the CSCD database as potential target miRNAs of DEcircRNAs from (GSE101586, GSE101684, and GSE112214). After that, these predicted target miRNAs were further screened by DEmiRNA from GSE135918. Furthermore, based on the TargetScan database^5^ (Wong and Wang, 2015), the miRTarBase database^6^ (Chou et al., 2016), and the miRDB database^7^ (Fromm et al., 2015), we predicted the interactions between miRNAs and mRNAs. Specifically, these mRNAs identified by the three databases were regarded as candidate mRNAs. The candidate mRNAs were further screened by DEmRNAs from GSE32863.

### Construction of the Novel ceRNA Network Related to LUAD

Relying on the identified DEcircRNAs, the predicted target miRNAs, and the predicted target mRNAs, we could determine the number and types of circRNA–miRNA pairs as well as the miRNA–mRNA pairs. Then, based on these circRNA–miRNA pairs and miRNA–mRNA pairs, the novel circRNA–miRNA–mRNA regulatory network related to LUAD was constructed and visualized via utilizing Cytoscape 3.6.1 software.

### GO and KEGG Functional Enrichment Analysis

For the purpose of exploring the molecular mechanism of genes in the novel ceRNA regulatory network related to LUAD, we implemented GO and KEGG functional enrichment analysis on basis of the Database for Annotation, Visualization and Integrated Discovery (DAVID)^8^ v6.8. In GO and KEGG functional enrichment analysis, *p* < 0.05 was given to imply a statistically significant difference. What is more, GO enrichment analysis consists of cellular component (CC), molecular function (MF), and biological process (BP), which, respectively, demonstrate the biological functions of genes at different levels (Zhang L. et al., 2019; Sun et al., 2020). KEGG pathway enrichment analysis is applied to assess the enrichment degree of genes in different pathways. DAVID is an online biological information database that integrates biological data and analysis tools and provides gene annotation information and protein data.

### Survival Analysis, RNA Extraction, and qRT-PCR

In order to select mRNAs associated with LUAD prognosis, we downloaded the mRNA expression profiles and the corresponding LUAD patient clinical information from the TCGA database and GEO database. Then, based on the survival algorithm package implemented by the R language, we conducted overall survival (OS) analysis experiments to verify each gene in the novel ceRNA regulatory network and plot Kaplan–Meier curves. The log-rank test was applied to achieve statistical verification (*p* < 0.05). The novel ceRNA hub regulatory network was constructed on the basis of these mRNAs related to LUAD prognosis. In addition, we utilized qRT-PCR to further validate the DEcircRNAs in the novel ceRNA hub regulatory network. According to instructions, we utilized Trizol reagent (Invitrogen, Carlsbad, CA, United States) to extract total RNA from fresh frozen LUAD tissues and adjacent non-tumor tissues. Then, Trans Script one-step guide DNA removal and complementary DNA synthesis super mix were used for the reverse transcription reaction. The primer sequences for PCR amplification were as follows: hsa_circ_0049271, forward, 5′-GCCCGGGAGTACATCTACAT-3′, reverse, 5′-TC AGTGGAGGCGTACATCAC-3′; hsa_circ_0004789, forward, 5′-TCTCGGTTGTGTTCGTCTTC-3′, reverse, 5′-CCATCAAC CACCACCTTAGC-3′; and hsa_circ_0043256, forward, 5′-TGTGGTGATCATGAATGGCTC-3′, reverse, 5′-TCACCCCG AATAGACAGCTC-3′. After qRT-PCR verification, we obtained the final ceRNA hub regulatory network associated with LUAD.

## Results

### Identification of DEcircRNAs, DEmiRNAs, and DEmRNAs in LUAD

**FIGURE 2:**
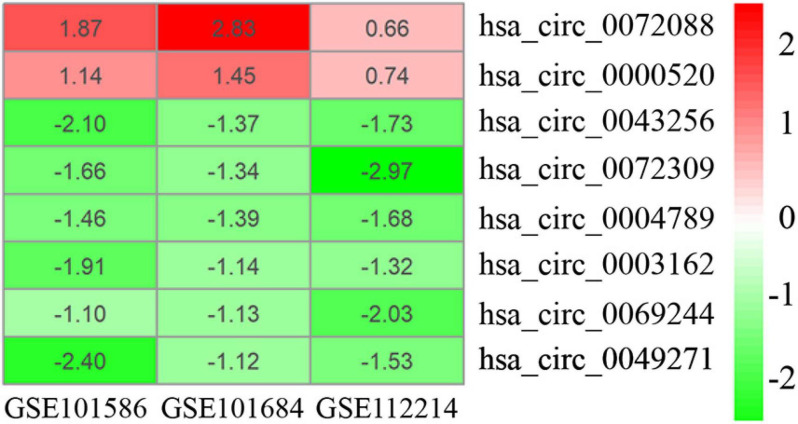
Heatmap of eight DEcircRNAs in three microarray datasets.

**FIGURE 3:**
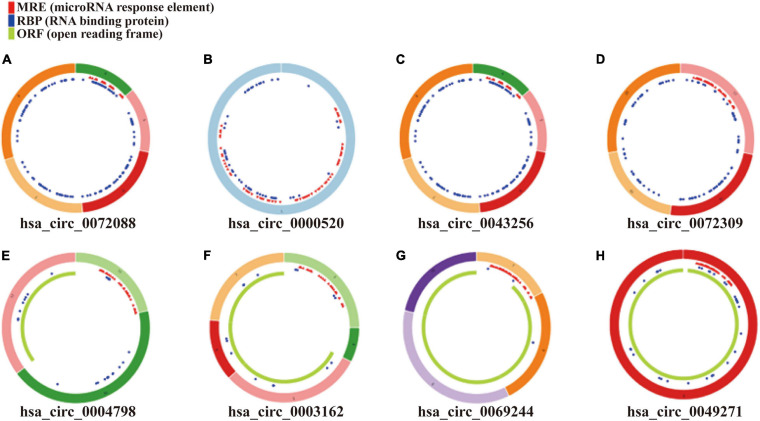
Structural patterns of eight DEcircRNAs. **(A)** hsa_circ_0072088, **(B)** hsa_circ_0000520, **(C)** hsa_circ_0043256, **(D)** hsa_circ_0072309, **(E)** hsa_circ_0004789, **(F)** hsa_circ_0003162, **(G)** hsa_circ_0069244, and **(H)** hsa_circ_0049271.

**FIGURE 4:**
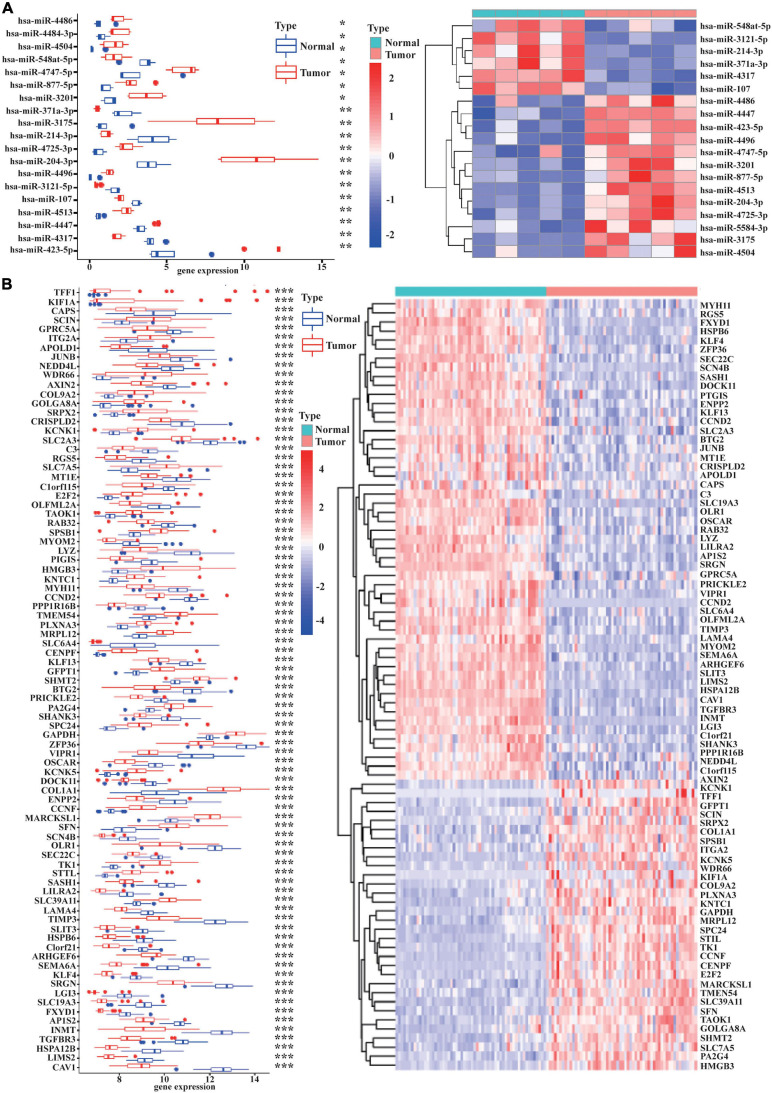
The 19 overlapped DEmiRNAs and the 85 overlapped DEmRNAs. The boxplot and heatmap of the 19 overlapped DEmiRNAs are shown in **(A)**. The 19 DEmiRNAs were selected through overlapping the 441 miRNAs binding to the 8 DEcircRNAs and the 237 DEmiRNAs identified in GSE135918. The expression levels of the 19 overlapped DEmiRNAs are described in **(A)**. The boxplot and heatmap of the 85 overlapped DEmRNAs are shown in **(B)**. The 85 DEmiRNAs were obtained by overlapping the 2,775 target genes binding to the 19 DEmiRNAs and the 956 DEmRNAs identified in GSE32863. The expression levels of the 85 overlapped DEmRNAs are displayed in **(B)**. [| log2(fold change)| > 1 and *p* < 0.05 were the cutoff criteria]. *p < 0.05, **p < 0.01, ***p < 0.001.

As displayed in Table 1, we have listed basic information of five microarray datasets (GSE101586, GSE101684, GSE112214, GSE135918, and GSE32863) regarding circRNAs, miRNAs, and mRNAs. Through integrating the data in GSE101586, GSE101684, and GSE112214, we identified eight DEcircRNAs (two upregulated circRNAs and six downregulated circRNAs, Figure 2). At the same time, the basic information and structural patterns of the eight DEcircRNAs are shown in Table 2 and Figure 3, respectively. Based on the CircInteractome and CSCD databases, we predicted 441 potential target miRNAs associated with the eight DEcircRNAs. Besides, a total of 237 DEmiRNAs (116 upregulated miRNAs and 121 downregulated miRNAs) were identified in GSE135918. Then, 19 overlapped DEmiRNAs (13 upregulated miRNAs and 6 downregulated miRNAs, Figure 4A) were acquired. Moreover, through taking advantages of TargetScan, miRTarBase, and miRDB databases, we obtained 2,775 target genes associated with the 19 overlapped DEmiRNAs. Simultaneously, a total of 956 DEmRNAs (339 upregulated mRNAs and 617 downregulated mRNAs) were identified in GSE32863. Eventually, we gained 85 overlapped DEmRNAs (32 upregulated mRNAs and 53 downregulated mRNAs, Figure 4B) in this research.

**TABLE 1:** Basic information of five microarray datasets from the GEO database.

Data source	Series	Platform	Author	Year	Area	Sample size (T/N)	No. of RNAs
circRNA	GSE101586	GPL19978	Qiu	2019	China	5/5	4,425
circRNA	GSE101684	GPL21825	Xu	2019	China	4/4	9,114
circRNA	GSE112214	GPL19978	Dong	2019	China	3/3	4,407
miRNA	GSE135918	GPL18058	Jiang	2020	China	5/5	3,551
mRNA	GSE32863	GPL6884	Selamat	2019	United States	58/58	48,803

**TABLE 2:** Basic characteristics of eight DEcircRNAs.

circRNA ID	Position	Genomic length	Strand	Best transcript	Gene symbol	Regulation
hsa_circ_0072088	chr5:32379220-32388780	9,560	−	NM_016107	ZFR	Up
hsa_circ_0000520	chr14:20811436-20811559	123	−	NR_002312	RPPH1	Up
hsa_circ_0043256	chr17:35604934-35609962	5,028	−	NM_198839	ACACA	Down
hsa_circ_0072309	chr5:38523520-38530768	7,248	−	NM_001127671	LIFR	Down
hsa_circ_0004789	chr17:62587201-62594608	7,407	−	NM_022739	SMURF2	Down
hsa_circ_0003162	chr7:33185853-33217203	31,350	+	NM_198428	BBS9	Down
hsa_circ_0069244	chr4:16587544-16760883	173,339	−	NM_001130834	LDB2	Down
hsa_circ_0049271	chr19:10610070-10610756	686	−	NM_203500	KEAP1	Down

### Construction of the ceRNA Regulatory Network in LUAD

**FIGURE 5:**
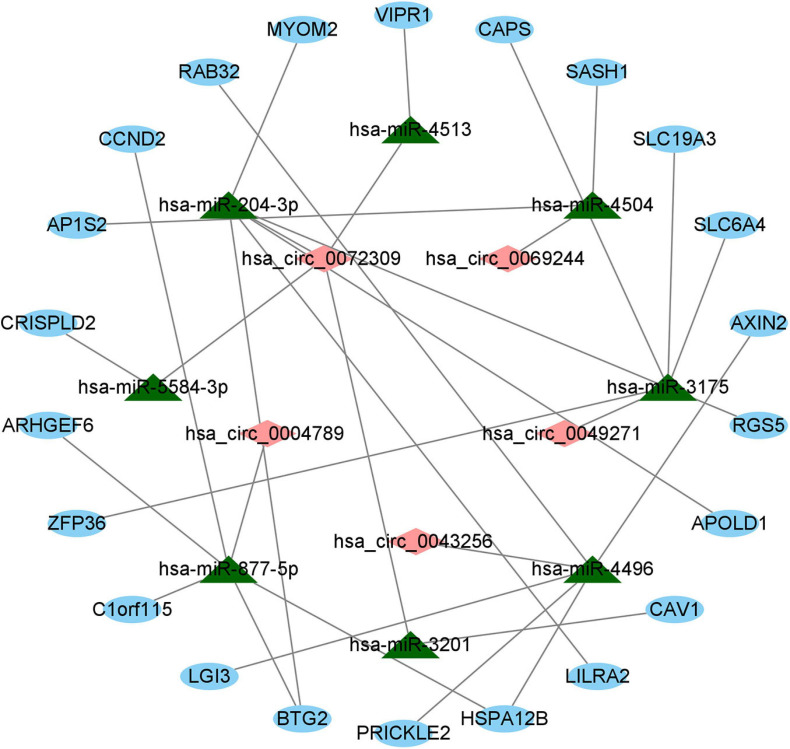
The ceRNA regulatory network on basis of circRNA–miRNA pairs and miRNA–mRNA pairs in LUAD. Red diamonds indicate circRNAs, green triangles indicate miRNAs, and blue ovals indicate mRNAs.

For the sake of further clarifying the mechanism by which circRNAs and miRNAs affect the occurrence and progression of LUAD, we established a ceRNA regulatory network and visualized the network. First, based on the predicted circRNA–miRNA pairs and miRNA–mRNA pairs, we identified eight miRNAs that have two types of relationships at the same time. Then, on the basis of eight DEcircRNAs, eight identified miRNAs and the predicted circRNA-miRNA pairs, a total of five circRNAs were obtained. Simultaneously, we received 22 mRNAs involved in the ceRNA regulatory network according to the 85 overlapped DEmRNAs, the 8 identified miRNAs, and the predicted miRNA–mRNA pairs. Furthermore, we also gained eight circRNA–miRNA pairs between the five identified circRNAs and the eight identified miRNAs, and 24 miRNA–mRNA pairs between the eight identified miRNAs and the 22 identified mRNAs. Ultimately, as described in Figure 5, a ceRNA regulatory network with the identified 32 edges (the 8 circRNA–miRNA edges and the 24 miRNA–mRNA edges) and the 35 identified nodes (5 circRNAs, 8 miRNAs, and 22 mRNAs) was constructed through utilizing the Cytoscape 3.8.0 software.

### Functional and Pathway Enrichment Analysis

**FIGURE 6:**
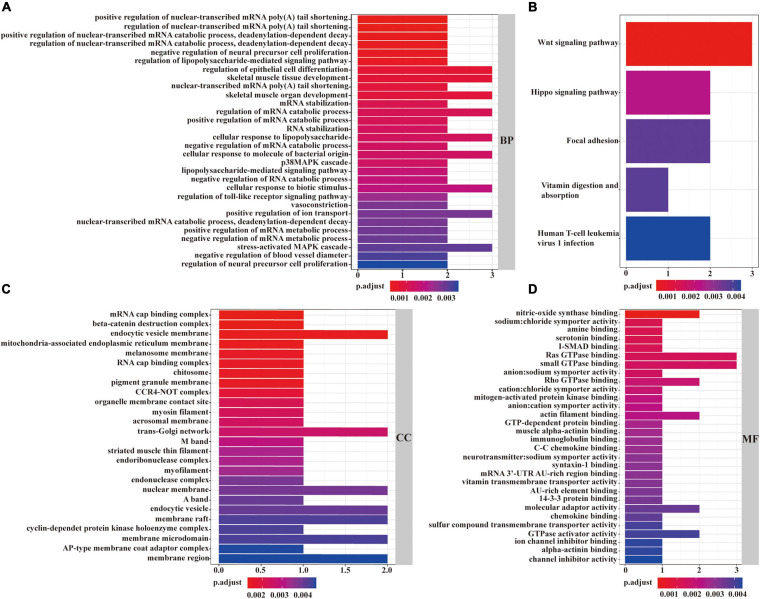
GO and KEGG functional enrichment analyses of mRNAs in the ceRNA network. The enrichment of biological processes is shown in **(A)**. The enrichment of cellular components was described in **(C)**. The enrichment of molecular functions is displayed in **(D)**. The enrichment analysis of KEGG pathways is shown in **(B)**. A *p* < 0.05 was considered to indicate a statistically significant difference.

As described in Figure 6A, in the view of BPs, the identified mRNAs in the ceRNA network were mainly enriched in “regulation of epithelial cell differentiation,” “skeletal muscle tissue development,” “regulation of mRNA catabolic process,” “cellular response to lipopolysaccharide,” and “cellular response to lipopolysaccharide.” According to CCs, these mRNAs were mainly enriched in “endocytic vesicle membrane,” “trans-Golgi network,” “mRNA cap binding complex,” “pigment granule membrane,” and “organelle membrane contact site” (Figure 6C). From the point of view of MFs, the mRNAs in the ceRNA network were mainly enriched in “nitric-oxide synthase binding,” “Ras GTPase binding,” “small GTPase binding,” “actin filament binding,” and “molecular adaptor activity” (Figure 6D). Besides, KEGG pathway analysis revealed the strong enrichment in “Wnt signaling pathway,” “Hippo signaling pathway,” “focal adhesion,” “vitamin digestion and absorption,” and “human T-cell leukemia virus 1 infection” (Figure 6B).

### Survival Analysis, qRT-PCR Validation, and the Construction of a ceRNA Hub Regulatory Network

**FIGURE 7:**
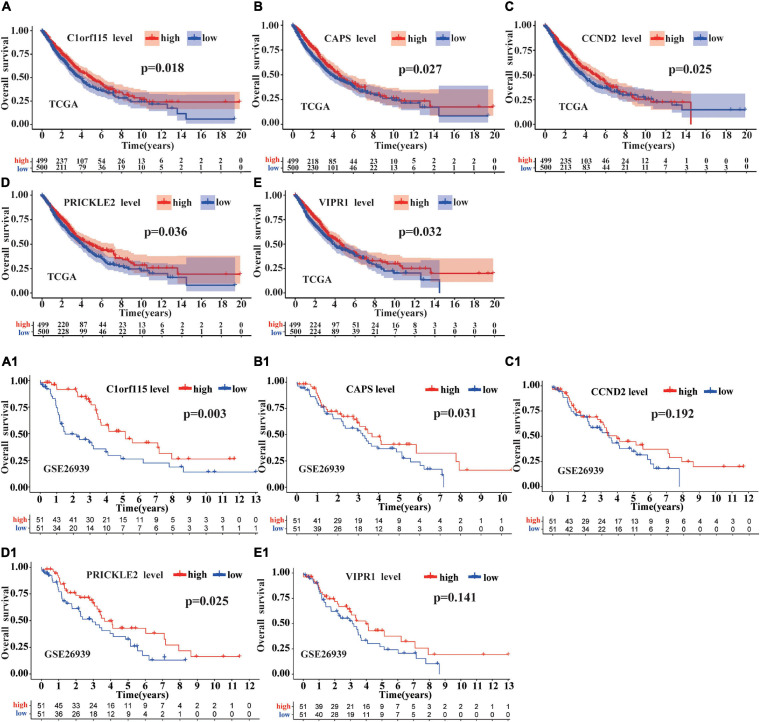
Survival analysis of mRNAs in ceRNA regulatory network. The survival prognosis of 22 mRNAs was analyzed based on the TCGA database. As shown in **(A)**, compared with the group with low C1orf115 expression, the group with high C1orf115 expression had a better prognosis in LUAD (*p* = 0.018). As displayed in Panels **(B–E)**, we have reached the same conclusion for CAPS, CCND2, PRICKLE2, and VIPR1 (*p* = 0.027, *p* = 0.025, *p* = 0.036, and *p* = 0.032). As depicted in **(A1), (B1), (C1), (D1),** and **(E1)**, based on GSE26939 array from the GEO database, only C1orf115, CAPS, and PRICKLE2 (*p* = 0.003, *p* = 0.031, and *p* = 0.025) have been verified to be consistent with the above conclusions.

**FIGURE 8:**
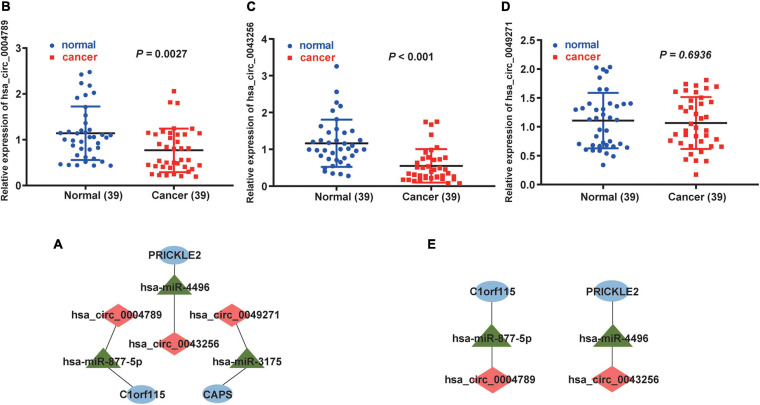
Construction of the final ceRNA hub regulatory network. **(A)** Construction of the ceRNA hub regulatory network based on the mRNAs associated with LUAD prognosis. As shown in **(B–D)**, expression of hsa_circ_0004789, hsa_circ_0043256, and hsa_circ_0049271 in 39 LUAD tissues and corresponding non-tumor tissues were detected by qRT-PCR, respectively (*p* = 0.0027, *p* < 0.001, and *p* = 0.6936). **(E)** After qRT-PCR verification, the final ceRNA hub regulatory network was constructed. In the ceRNA hub regulatory network, red diamonds indicate circRNAs, green triangles indicate miRNAs, and blue ovals indicate mRNAs.

First, we downloaded 1,038 LUAD samples with expression profiles and complete clinical information from the TCGA database. After filtering out samples without survival information, we kept 999 samples. According to the median expression value, we divided the samples into the high expression group (499 samples) and the low expression group (500 samples). Then, the survival analysis was carried out for each mRNA (a total of 22 mRNAs in circRNA–miRNA–mRNA regulatory network). We observed that five mRNAs (C1orf115, CAPS, CCND2, PRICKLE2, and VIPR1) were associated with prognosis in LUAD patients (Figures 7A–E). To further investigate the significance of the five mRNAs in prognosis prediction of LUAD, we obtained GSE26939 array from the GEO database (51 samples in the high expression group and 51 samples in the low expression group) and conducted the survival analysis experiment. As a result, three mRNAs (C1orf115, CAPS, and PRICKLE2) were identified to be related to the prognosis prediction of LUAD (Figures 7A1–E1). The above analysis showed that C1orf115 was significantly associated with prognosis in LUAD patients (*p* = 0.018 in TCGA and *p* = 0.003 in GSE26939). Besides, from the statistical perspective, CAPS and PRICKLE2 had certain relationships with prognosis prediction of LUAD, but the observed differences in median survival between the high expression group and the low expression group were not prominent. Considering the impact of the sample size on statistical analysis, and the significant differences in expression levels of CAPS and PRICKLE2 between normal tissues and LUAD tissues, we accepted that CAPS and PRICKLE2 were related to the prognosis prediction of LUAD from the biological perspective. On basis of the three overlapped mRNAs (C1orf115, CAPS, and PRICKLE2), we constructed a circRNA–miRNA–mRNA hub regulatory network (Figure 8A). As shown in Figures 8B–D, qRT-PCR result confirmed that hsa_circ_0049271 was not significantly differentially expressed between LUAD tissues and adjacent non-tumor tissues (*p* = 0.6936). Thus, we held the opinion that hsa_circ_0049271/hsa-miR-3175/CAPS axis might not regulate the LUAD occurrence and progression. The independent regulatory mechanism of hsa_circ_0049271 downstream network needs further verification. In this study, we removed hsa_circ_0049271/hsa-miR-3175/CAPS axis and kept hsa_circ_0004789/hsa-miR-877-5p/C1orf115 and hsa_circ_0043256/hsa-miR-4496/PRICKLE2 axes verified by qRT-PCR to obtain the final ceRNA hub regulatory network (Figure 8E).

## Discussion

CircRNAs are a special type of non-coding RNAs with a circular biological structure (Zhang L. et al., 2020). Due to the closed loop structure, circRNAs are not easily affected by the RNA exonuclease and have the high stability in the human body (Cao et al., 2021). CircRNAs have been observed in various cancers and can affect the occurrence and progression of different cancers, such as colorectal cancer (Chen J. et al., 2020), gastric cancer (Chen H. et al., 2020), and liver cancer (Tang et al., 2020). Evidence has shown that circRNAs could indirectly regulate protein-coding genes through reducing the expression level of related miRNAs. The latest research announced circRNAs might play a decisive role in the occurrence and progression of tumors, and some circRNAs could become important biomarkers of cancer prognosis.

Lung adenocarcinoma, a type of NSCLC, is the most common kind of lung cancer, accounting for approximately 40% in all types of lung cancers (Xu J.Y. et al., 2020). LUAD is frequently detected at the metastatic stage with prevalence in the brain, bones, and respiratory system (Kim et al., 2020). The current treatments for LUAD are mainly surgery, radiotherapy, chemotherapy, and immunotherapy, but the prognosis of patients is still unsatisfactory. Due to their stable structure, circRNAs are promised to become a new strategy for LUAD-targeted therapy. Xu et al. observed that HMGA2 could regulate circASPH to promote tumor growth in patients with LUAD (Xu L. et al., 2020). The study of Lu G.J. et al. (2020) revealed that hsa_circ_0001715 was upregulated in LUAD and had expectation to become a novel diagnostic and prognostic marker. Besides, Wang et al. (2020) demonstrated that based on the miR-138/Sirt1 pathway, hsa_circ_0006571 could promote the migration and invasion of LUAD cells. Although these studies indicate that circRNAs are abnormally expressed in LUAD and have associations with LUAD occurrence and progression, the specific functional pathway and molecular mechanism of circRNAs in LUAD are still unclear.

To explore the role of circRNAs in LUAD, we constructed a ceRNA regulatory network based on data mining, bioinformatics methods, and public databases. GO analysis displayed that genes in our ceRNA regulatory network were involved in the regulation of beta-catenin destruction complex, trans-Golgi network, and small GTPase binding. Some studies declared that non-coding RNAs could regulate the progression cancer cells via regulating the Wnt/beta-catenin signal. Not only that, the Wnt/beta-catenin pathway could also promote cancer stem cell self-renewal, metastasis, and chemoresistance in all types of epithelial ovarian cancer (Nguyen et al., 2019). Bao et al. (2015) proved that via interfering with trans-Golgi network trafficking, small molecule schweinfurthins selectively inhibited cancer cell proliferation and mTOR/AKT signaling. Additionally, in view of the intimate crosstalk of Rho/Rac1/Rac1b and TGF-beta signaling in tumor cell responses, targeting specific Rho GTPases might allow for selective interference with prooncogenic TGF-beta responses to aid in anticancer treatments (Ungefroren et al., 2018). KEGG analysis indicated that mRNAs in this ceRNA regulatory network was enriched in Wnt signaling pathway and Hippo signaling pathway, which have been confirmed by many studies to participate in cancer progression (Ansari et al., 2019; Flanagan et al., 2019). Besides, Namani et al. revealed that NRF2 could regulate the occurrence and progression of NSCLC by regulating genes involved in the focal adhesion pathway (Namani et al., 2019). It has not been reported that “vitamin digestion and absorption” and “human T-cell leukemia virus 1 infection” can regulate cancer progression, and further investigations are needed. All in all, the above evidence suggest that these circRNAs from this ceRNA regulatory network may act as a key role in LUAD occurrence and progression.

Based on the TCGA and GEO databases, we analyzed the correlation between expression levels of 22 mRNAs in the ceRNA regulatory network and OS of LUAD patients. As a result, three mRNAs (C1orf115, CAPS, and PRICKLE2) were identified to be associated with the prognosis of LUAD patients. Then, based on mRNAs related to LUAD prognosis, we constructed a novel ceRNA hub regulatory network. Hsa_circ_0004789 and hsa_circ_0043256 were verified by qRT-PCR to be differentially expressed between LUAD tissues and adjacent non-tumor tissues. Two miRNAs (hsa-miR-877-5p and hsa-miR-4496) were also included in final ceRNA hub regulatory network. It has been reported that the loss of C1orf115 can lead to the resistance to five anticancer drugs, and the low expression of C1orf115 is related to the poor prognosis of many cancers (Lau et al., 2020). Besides, Senchenko et al. (2013) proved that PRICKLE2 was significantly related to the prognosis of various cancers. What is more, the specific functional pathway and molecular mechanism of the two identified genes in final novel ceRNA hub regulatory network need to be further studied.

The significance of this study lies in not only constructing a novel ceRNA hub regulatory network through utilizing GEO database but also screening out mRNAs associated with LUAD prognosis via integrating clinical data in TCGA and GEO databases. However, this research also has some limitations because it mainly relies on analysis of sequencing data and public clinical data and requires further experimental explorations.

In conclusion, based on publicly available sequencing and clinical data, we identified differentially expressed circRNAs, miRNAs, and mRNAs and constructed a circRNA-related regulatory network. Through utilizing TCGA and GEO databases, we identified three mRNAs related to LUAD prognosis and built a novel ceRNA hub regulatory network. Two circRNAs in final ceRNA hub regulatory network were validated by qRT-PCR to be differentially expressed between LUAD tissues and adjacent non-tumor tissues. This study may provide new insights into the pathogenesis of LUAD and reveal potential therapeutic targets.

## Data Availability Statement

The datasets presented in this study can be found in online repositories. The names of the repository/repositories and accession number(s) can be found in the article/Supplementary material.

## Ethics Statement

The project was granted approval by the Ethics Committee of the Affiliated Hospital of Xuzhou Medical University. The patients/participants provided their written informed consent to participate in this study.

## Author Contributions

HZ and XL designed the overall idea of this study, conceived the experiments, analyzed the data, prepared the figures and tables, and authored the drafts of the manuscript. XZ and QS collected the data from the TCGA and GEO dataset and performed the experiments. QY and YX supervised this study and reviewed the drafts of the manuscript. All authors read and approved the final draft.

## Conflict of Interest

The authors declare that the research was conducted in the absence of any commercial or financial relationships that could be construed as a potential conflict of interest.

## Footnotes

1 http://www.ncbi.nlm.nih.gov/geo

2 https://tcga-data.nci.nih.gov/tcga/

3 https://circinteractome.nia.nih.gov/

4 https://gb.whu.edu.cn/CSCD/

5 http://www.targetscan.org/vert_72/

6 http://mirtarbase.cuhk.edu.cn/php/index.php

7 http://mirdb.org/

8 http://david.abcc.ncifcrf.gov/
